# Lemierre’s syndrome

**DOI:** 10.1186/1865-1380-6-40

**Published:** 2013-10-23

**Authors:** Wesley Eilbert, Nitin Singla

**Affiliations:** 1 Department of Emergency Medicine, University of Illinois, College of Medicine, 1819 West Polk Street, Room 471 CME, Chicago, IL 60612, USA

**Keywords:** Lemierre, Lemierre’s syndrome, Lemierre’s disease, Lemierre syndrome, Fusobacterium necrophorum, Fusobacterium, Septic thrombophlebitis

## Abstract

Lemierre’s syndrome is a condition characterized by thrombophlebitis of the internal jugular vein and bacteremia caused by primarily anaerobic organisms, following a recent oropharyngeal infection. This has been an uncommon illness in the era of antibiotic therapy, though it has been reported with increasing frequency in the past 15 years. Lemierre’s syndrome should be suspected in young healthy patients with prolonged symptoms of pharyngitis followed by symptoms of septicemia or pneumonia, or an atypical lateral neck pain. Diagnosis is often confirmed by identification of thrombophlebitis of the internal jugular vein and growth of anaerobic bacteria on blood culture. Treatment involves prolonged antibiotic therapy occasionally combined with anticoagulation. We review the literature and a case of a child with Lemierre’s syndrome.

## Review

In 1936, Andre Lemierre published a case series of 20 patients with a syndrome characterized by a history of recent oropharyngeal infection, clinical or radiological evidence of internal jugular (IJ) venous thrombosis, and anaerobic septicemia caused primarily by *Bacillus funduliformis* (now known as *Fusobacterium necrophorum*) [1]. Lemierre classified this syndrome as “anaerobic postanginal sepsis” because of the onset of sepsis occurring shortly after the patients had experienced a sore throat. It was not until the 1980s that anaerobic postanginal sepsis was routinely referred to as Lemierre’s syndrome [2]. With the introduction of antibiotics in the 1940s and their widespread use for streptococcal pharyngitis, the incidence of Lemierre’s syndrome fell dramatically. In fact, some authors in the 1980s and 1990s referred to it as a “forgotten disease” [3-5]. For reasons that are not clear, there has been an increase in the reporting of Lemierre’s syndrome since the late 1990s [6-8]. We report here a case of Lemierre’s syndrome in an adolescent and review the literature on this uncommon condition.

## Case report

### History

A previously healthy 13-year-old boy was brought to our emergency department (ED) by his mother complaining of right-sided throat pain present for 5 days. The pain radiated to his right ear and down the right side of his neck. It was aggravated by swallowing as well as flexion, extension and rotation of his neck. His mother reported a tactile fever also present for 5 days, though noted no change in the child’s voice. The patient had been evaluated by his pediatrician 2 days before coming to the ED and was prescribed amoxicillin for suspected streptococcal pharyngitis. His mother was concerned as the throat pain had worsened despite the antibiotics.

### Physical exam

The patient arrived to the ED with a temperature of 37.8°C, though was found to have a temperature of 38.1°C 1 h later. He appeared to be in no distress, and examination of the heart, lungs, abdomen and skin was unremarkable. Head examination revealed normal tympanic membranes and external auditory canals bilaterally and no mastoid tenderness or edema. His oropharynx was noted to be nonerythematous and without exudates, and his uvula was midline. His neck was supple with enlarged, tender lymph nodes palpable in the right anterior cervical chain.

### Diagnostic testing

**Figure 1:**
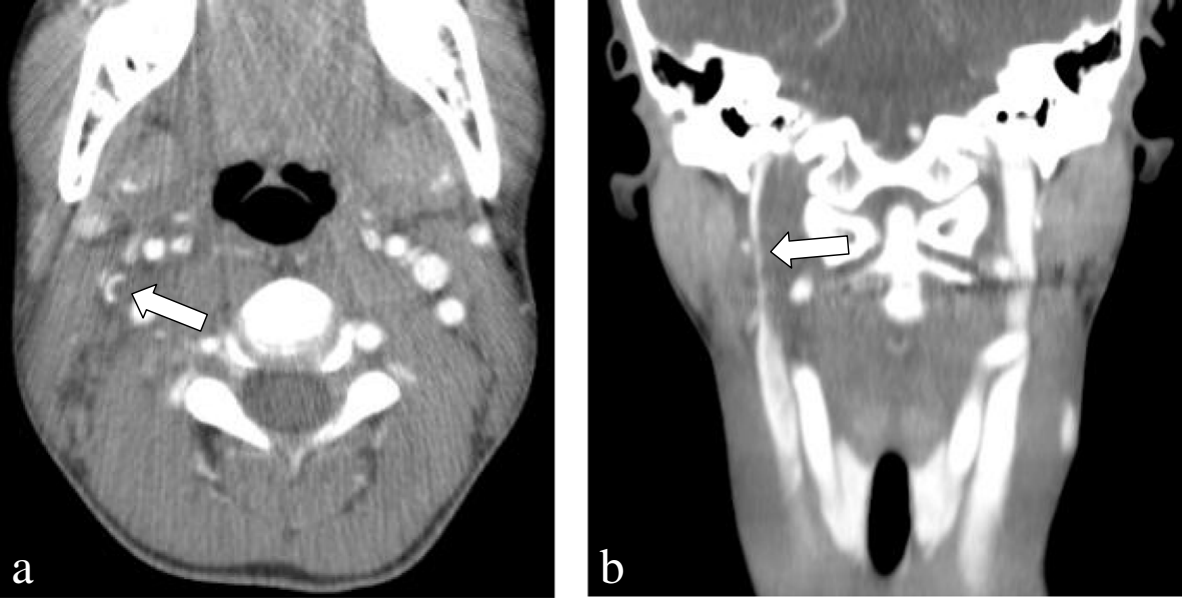
**Computed tomography of the neck with intravenous contrast demonstrating partial occlusion of the right internal jugular vein (*arrows*) seen on the transverse (a) and coronal (b) views.**

A complete blood count included a white blood cell count of 9,400 cells/mcL with 69% neutrophils. Serum electrolytes were all within normal limits, and urinalysis was negative for signs of infection. Computed tomography (CT) of the neck with intravenous (IV) contrast was obtained because of concern for a possible deep space abscess. While the CT showed no abscess, an opacification of the right IJ vein was seen extending from the jugular foramen to the common jugular confluence, suspicious for high-grade partial occlusion (Figure 1).

### Treatment

The patient was given a dose of IV ampicillin/sulbactam for suspected septic thrombophlebitis of the right IJ vein and transferred to a nearby children’s hospital for admission and further treatment. During his 5-day hospital course, the patient had no signs of metastatic infection. Two sets of blood cultures drawn on hospital days 1 and 5 grew no organisms. Workup for the presence of a hypercoagulable state was negative, and the patient was anticoagulated with enoxaparin. He was discharged home to complete a 6-week course of antibiotics and anticoagulation. At a clinic visit 1 month later, the patient was doing well with only a complaint of mild weight loss.

## Discussion

### Epidemiology and pathophysiology

In all major case series and reviews, Lemierre’s syndrome is primarily an affliction of previously healthy children, adolescents and young adults [2,8-17]. In a review of 114 patients, Karkos et al. [17] found that most cases presented in the 2nd decade of life (51%), followed by the 3rd decade (20%) and then the 1st decade (8%). There is no clear gender predominance with Lemierre’s syndrome, and most cases are diagnosed during the “sore throat season” in the late winter and early spring [18]. While not uncommon in the preantibiotic era, Lemierre’s syndrome is now a rare condition with an incidence of 3.6 cases per 1 million per year [8]. Several authors have noted an increase in the number of cases of Lemierre’s syndrome since the late 1990s [6-8]. Theories as to the cause of this slight resurgence include an increased awareness of the syndrome, population changes and the more judicious use of antibiotics for the treatment of streptococcal pharyngitis [2,7,14].

*Fusobacterium* species, most commonly *Fusobacterium necrophorum,* are responsible for the majority of bacteremia in cases of Lemierre’s syndrome [7,9-13,15-17,19,20]. Up to one third of patients will have a polymicrobial bacteremia, with anaerobic streptococci and other miscellaneous gram-negative anaerobes frequently present [9,17]. *F. necrophorum* is a gram-negative anaerobic rod that is part of the normal flora of the oropharynx. It is not known what causes this typically noninvasive organism to penetrate mucosal surfaces, though some authors have proposed an alteration in the pharyngeal mucosa by a viral or bacterial pharyngitis might play a role. Several cases of Lemierre’s syndrome preceded by infectious mononucleosis have been reported [21,22]. The palatine tonsils and peritonsillar tissue are the primary sources of infection in most cases [2,7,8,10,11,13,15-17,19]. Other primary sources of infection include the lungs, middle ear, mastoid, teeth and sinuses [8,10,13,15,17,19]. Following the primary infection, there is local invasion of the lateral pharyngeal space and septic thrombophlebitis of the IJ vein.

Metastatic infections following the IJ thrombophlebitis occur in 63%-100% of patients [9,13,15,16,19]. The lungs are by far the most common site of metastatic infection in Lemierre’s syndrome, followed by the major joints. Metastatic infections of the liver, muscle, pericardium, brain and skin have also been described [9,12,15,19].

### Clinical presentation

The common presenting symptoms of Lemierre’s syndrome are listed in Table 1[2,9-12,15,17]. Sore throat is the most common symptom in all major series and reviews [2,7,10,15,17,19]. The onset of the sore throat typically precedes all other symptoms by 4–5 days, though this interval may be up to 12 days [12,18]. Some patients will have complete resolution of pharyngitis symptoms prior to the onset of symptoms from the IJ thrombophlebitis or metastatic infection. The neck pain with Lemierre’s syndrome is typically unilateral and may be aggravated by turning the head away from the involved side as a consequence of irritation of the sternocleidomastoid muscle [15]. Since the lungs are the most common site of metastatic infection, chest pain and pulmonary complaints are the most consistent indicators of metastatic disease.

**Table 1:** Common presenting symptoms of Lemierre’s syndrome

Sore throat	Pleuritic chest pain
Neck mass	Dyspnea
Neck pain	Cough/hemoptysis
Ear pain	Bone/joint pain
Dental pain	Abdominal pain

The physical exam findings of Lemierre’s syndrome are variable and depend on the extent of metastatic disease (Table 2). Fever is the most common physical finding reported, present in 92% to 100% of cases, followed by pharyngitis or peritonsillar abscess and neck mass [2,7,9,10,12-18,23,24]. A mass in the neck may be palpable at the angle of the jaw or along the anterior margin of the sternocleidomastoid muscle. Frequently, a mass in these locations is mistaken for enlarged lymph nodes [18,23]. Chirinos et al. [15], in a review of 109 cases of Lemierre’s syndrome, found 52% of patients had a swollen or tender neck, while 48% had no significant neck findings. For reasons that are not clear, a significant percentage of patients (11%-49%) with Lemierre’s syndrome will be jaundiced [10,12,13]. Not surprisingly, shock is a late finding in the disease process and a predictor of mortality.

**Table 2:** Common physical exam findings of Lemierre’s syndrome

Fever	Septic arthritis (most commonly hip or knee)
Pharyngitis/peritonsillar abscess	Jaundice/hepatomegaly
Neck mass/tenderness	Cranial nerve 10, 11, 12 palsies
Anterior cervical lymphadenopathy	Shock
Trismus	

### Diagnosis

Lemierre [1] in his original case series stated: “The appearance and repetition several days after the onset of a sore throat (and particularly of a tonsillar abscess) of several pyrexial attacks with an initial rigor, or still more certainly the occurrence of pulmonary infarcts and arthritic manifestations, constitute a syndrome so characteristic that mistake is almost impossible”. While this may have been true in the preantibiotic era, most ED physicians today will likely never encounter a patient with this disease. Indeed, Alvarez et al. [19] reported an average delay of 5 days from the time of admission until the correct diagnosis was made.

Karkos et al. [17] found that chest x-ray was the first-line investigation in 92% of patients ultimately diagnosed with Lemierre’s syndrome. This is probably a reflection of the high percentage of patients with metastatic lung infection. Lung lesions typically begin as multiple, usually nodular infiltrates that progress rapidly to cavitary lesions and are frequently accompanied by pleural effusions [23]. Not surprisingly, a significant number of patients with Lemierre’s syndrome are treated initially for pneumonia or right-sided staphylococcal endocarditis [12,18]. Between 13% and 27% of patients with Lemierre’s syndrome will undergo arthrocentesis because of symptoms of septic arthritis [9,10,12,13,16]. Joint fluid analysis will be similar to other causes of septic arthritis, though several authors have noted the aspirated pus to have a particularly foul odor [16].

The identification of thrombophlebitis of the IJ vein is the first hard evidence to suggest Lemierre’s syndrome in many patients [18]. It is likely true that, as with our patient, these imaging studies are ordered in the majority of patients to assess for deep space infections of the neck and not IJ vein thrombophlebitis. Duplex ultrasonography, CT and magnetic resonance imaging have all been used for IJ vein imaging, with CT being most commonly requested in patients with Lemierre’s syndrome [17]. Many authors consider contrast-enhanced CT as the preferred study in this setting as it allows for visualization of surrounding structures and is the most readily available [2,13,19,25].

Another key factor in the diagnosis of Lemierre’s syndrome is the growth of characteristic anaerobic bacteria from blood culture. This defining feature has been reported in the overwhelming majority of cases [9,10,12,13,16]. Unfortunately, this process may take from 2 to 7 days to occur [14], and, as with our patient, may be suppressed by the previous administration of antibiotics.

Ultimately, the diagnosis of Lemierre’s syndrome may be made only when considered in the appropriate clinical setting. Wright et al. [2] suggested increased suspicion for this illness when any of the following are true: pharyngitis that does not resolve in 3 to 5 days; pharyngitis followed by systemic or respiratory symptoms such as fever, chills, rigors or dyspnea; pharyngitis associated with lateral cervical pain and dysphagia; and pharyngitis followed by sepsis or multiple pulmonary abscesses.

### Morbidity and mortality

Lemierre [1] reported a mortality rate of 90% from his original case series in the preantibiotic era. Studies from the modern era have reported mortality rates from 0%-18% [8-10,12,13,15,16]. Disseminated intravascular coagulation has been reported in 3%-9% of cases [10,12,16]. Thrombosis may propagate from the IJ vein inferiorly into the subclavian vein or superiorly into the cavernous, sigmoid or transverse sinuses [7,14,16-18]. Meningitis may also complicate up to 3% of cases [12,13,16].

### Treatment

Prolonged antibiotic therapy constitutes the mainstay of treatment of Lemierre’s syndrome in the modern era. Since no controlled clinical trials exist to identify an optimal antibiotic regimen, decisions must be based on known in vitro sensitivities together with anecdotal clinical evidence. While penicillin monotherapy has been used in the past, more recent antimicrobial studies have indicated beta-lactamase activity acquired by many *F. necrophorum* strains [26]. Suggested empiric antibiotic options for the treatment of Lemierre’s syndrome are listed in Table 3[2,8,14,18,19,25,27]. It should be noted that some authors have recommended against the use of monotherapy with metronidazole because of the frequent occurrence of mixed infection with other oral flora [14]. The duration of antibiotic therapy should be from 2 to 6 weeks [2,18,25].

**Table 3:** Suggested empiric antibiotic options for Lemierre’s syndrome

Metronidazole	Ampicillin – sulbactam
Clindamycin	Ticarcillin – clavulanate
Penicillin plus metronidazole	Imipenem

The use of anticoagulation is controversial, and no controlled studies exist. Case series have reported 21%-30% of patients are treated with anticoagulation [17,18,28]. A few authors have advocated for the use of anticoagulants in all cases of Lemierre’s syndrome [20,24]. Others have recommended anticoagulation only if thrombosis extends into the cerebral sinuses or if there has been no improvement in symptoms with antibiotic therapy alone [8,14,18,25,29].

Surgical treatment of Lemierre’s syndrome may involve drainage of abscesses in the neck, most commonly peritonsillar or lateral pharyngeal abscesses. In the pre-antibiotic era, IJ vein ligation or excision was frequently performed to prevent septic embolization [30]. In the modern era, this drastic measure is taken only when there is evidence of continued septic embolization despite appropriate medical therapy [15,18,19,25,27].

## Conclusion

Lemierre’s syndrome occurs primarily in young, otherwise healthy individuals and is characterized by a history of recent oropharyngeal infection, clinical or radiological evidence of IJ venous thrombosis and anaerobic bacteremia caused primarily by *F. necrophorum*. This is a rare illness in the modern era of antibiotic therapy, though it has been reported with increasing frequency in the twenty-first century. Lemierre’s syndrome should be suspected in young, healthy patients with prolonged symptoms of pharyngitis followed by symptoms of septicemia or pneumonia, or an atypical lateral neck pain. Diagnosis is often confirmed by the identification of IJ vein thrombophlebitis by an imaging study and growth of anaerobic bacteria on blood culture. Prolonged antibiotic therapy is the cornerstone of treatment, occasionally combined with anticoagulation.

## Competing interests

We have no competing interests to report.

## Authors’ contributions

NS provided the information for the case report section of the article and performed the preliminary literature search. WE performed the definitive literature search and wrote the manuscript. Both authors read and approved the final manuscript.

## References

[B1] Lemierre A On certain septicemias due to anaerobic organisms Lancet 1936 6 701 703

[B2] Wright WF Shiner CN Ribes JA Lemierre syndrome South Med J 2012 6 5 283 288 10.1097/SMJ.0b013e31825581ef 22561543

[B3] Moore-Gillon J Lee TH Eykyn SJ Phillips I Necrobacillosis: a forgotten disease BMJ 1984 6 6429 1526 1527 10.1136/bmj.288.6429.1526 6426626 PMC1441150

[B4] Weesner CL Cisek JEL Lemierre syndrome: the forgotten disease Ann Emerg Med 1993 6 2 256 288 10.1016/S0196-0644(05)80216-1 8427443

[B5] Koay CB Heyworth T Burden P Lemierre syndrome: a forgotten complication of acute tonsillitis J Laryngol Otol 1995 6 6 657 661 7561477 10.1017/s0022215100130956

[B6] Brazier JS Hall V Yusef E Duerden B *Fusobacterium necrophorum* infections in England and Wales 1990–2000 J Med Microbiol 2002 6 3 269 272 11871622 10.1099/0022-1317-51-3-269

[B7] Ramirez S Hild TG Rudolph CN Sty JR Kehl SC Havens P Henrickson K Chusid MJ Increased diagnosis of Lemierre’s syndrome and other *Fusobacterium necrophorum* infections at a children’s hospital Pediatrics 2003 6 5 e380 e385 10.1542/peds.112.5.e380 14595080

[B8] Hagelskjaer Kristensen L Prag J Lemierre’s syndrome and other disseminated *Fusobacterium necrophorium* infections in Denmark: a prospective epidemological and clinical survey Eur J Clin Microbiol Infect Dis 2008 6 9 779 789 10.1007/s10096-008-0496-4 18330604 PMC7102232

[B9] Hagelskjaer LH Prag J Malczyski J Kristensen JH Incidence and clinical epidemiology of necrobacillosis, including Lemierre’s syndrome in Denmark 1990–1995 Eur J Clin Microbiol Infect Dis 1998 6 8 561 565 9796654 10.1007/BF01708619

[B10] Moreno S Garcia Altozano J Pinilla B Lopez JC de Quiros B Ortega A Bouza E Lemierre’s disease: postanginal bacteremia and pulmonary involvement caused by *Fusobacterium necrophorum* Rev Infect Dis 1989 6 2 319 324 10.1093/clinids/11.2.319 2649965

[B11] Jones JW Riordan T Morgan MS Investigation of postanginal sepsis and Lemierre’s syndrome in the south west peninsula Commun Dis Public Health 2001 6 4 278 282 12109395

[B12] Leugers CM Clover R Lemierre syndrome: postanginal sepsis J Am Board Fam Pract 1995 6 5 384 391 7484226

[B13] Sinave CP Hardy GJ Fardy PW The Lemierre syndrome: thrombophlebitis of the internal jugular vein secondary to oropharyngeal infection Medicine (Baltimore) 1989 6 2 85 94 2646510

[B14] Riordan T Wilson M Lemierre’s syndrome: more than a historical curiosa Postgrad Med J 2004 6 944 328 334 10.1136/pgmj.2003.014274 15192164 PMC1743018

[B15] Chirinos JA Lichstein DM Garcia J Tamariz LJ The evolution of Lemierre syndrome Medicine (Baltimore) 2002 6 6 458 465 10.1097/00005792-200211000-00006 12441902

[B16] Eykyn SJ Necrobacillosis Scand J Infect Dis 1989 6 41 46 2685991

[B17] Karkos PD Asrani S Karkos CD Leong SC Theochari EG Alexopoulou TD Assimakopoulos AD Lemierre’s syndrome: a systematic review Laryngoscope 2009 6 8 1552 1559 10.1002/lary.20542 19554637

[B18] Riordan T Human Infection with *Fusobacterium necrophorum* (necrobacillosis), with a focus on Lemierre’s syndrome Clin Microbiol Rev 2007 6 4 622 659 10.1128/CMR.00011-07 17934077 PMC2176048

[B19] Alvarez A Schreiber JR Lemierre’s syndrome in adolescent children - anaerobic sepsis with internal jugular vein thrombophlebitis following pharyngitis Pediatrics 1995 6 354 359 7630700

[B20] Goldenhagen J Alford BA Prewitt LH Thompson L Hostetter MK Suppurative thrombophlebitis of the internal jugular vein: report of three cases and review of the pediatric literature Pediatric Infect Dis J 1988 6 6 410 414 10.1097/00006454-198806000-00008 3293000

[B21] Dagan R Powell KR Postanginal sepsis following infectious mononucleosis Arch Intern Med 1987 6 9 1581 1583 10.1001/archinte.1987.00370090059012 3632166

[B22] Gruber B Mhoon EE Bilateral deep space neck abscesses complicating infectious mononucleosis Otolaryngol Head Neck Surg 1987 6 1 66 68 3112687 10.1177/019459988709700112

[B23] Burden P *Fusobacterium necrophorum* and Lemierre’s syndrome J Infect 1991 6 3 227 231 10.1016/0163-4453(91)92684-W 1753131

[B24] Carlson ER Bergamo DF Coccia CT Lemierre’s syndrome: two cases of a forgotten disease J Oral Maxillofac Surg 1994 6 1 74 78 10.1016/0278-2391(94)90019-1 8263648

[B25] Lustig LR Cusick BC Cheung SW Lee KC Lemierre’s syndrome: two cases of postanginal sepsis Otolaryngal Head Neck Surg 1995 6 6 767 772 10.1016/S0194-5998(95)70192-3 7777368

[B26] Appelbaum PC Spangler SK Jacobs MR Beta-lactamase production and susceptibilities to amoxicillin, amoxicillin-clavulanate, ticarcillin, ticarcillin-clavulanate, cefoxitin, imipenem, and metronidazole of 320 non-Bacteroides fragilis Bacteroides isolates and 129 fusobacteria from 28 U.S. centers Antimicrob Agents Chemother 1990 6 8 1546 1550 10.1128/AAC.34.8.1546 2221864 PMC171870

[B27] Golpe R Marin B Alonso M Lemierre’s syndrome (necrobacillosis) Postgrad Med J 1999 6 881 141 144 10448489 10.1136/pgmj.75.881.141 PMC1741175

[B28] Armstrong AW Spooner K Sanders JW Lemierre’s syndrome Curr Infect Dis Report 2000 6 2 168 173 10.1007/s11908-000-0030-z 11095853

[B29] Hoehn KS Lemierre’s syndrome: the controversy of anticoagulation Pediatrics 2005 6 5 1415 1416 15867055 10.1542/peds.2005-0138

[B30] Lemierre A Gregoire R Laporte A Couvelaire R Les aspects chirurgicaux des infections a *Bacillus funduliformis* Acad Med 1938 6 352 359

